# Application of Digital Technology in Prosthetic Treatment for a Cleft Lip and Palate Patient: A Novel Dental Technique

**DOI:** 10.7759/cureus.87298

**Published:** 2025-07-04

**Authors:** Mariko Hattori, Yuichi Yamatani, Mai Murase, Mihoko Haraguchi, Yuka Sumita, Noriyuki Wakabayashi

**Affiliations:** 1 Department of Advanced Prosthodontics, Institute of Science Tokyo, Tokyo, JPN; 2 Dental Laboratory, Institute of Science Tokyo Hospital, Tokyo, JPN; 3 Department of Partial and Complete Denture, The Nippon Dental University, School of Life Dentistry at Tokyo, Tokyo, JPN

**Keywords:** cleft lip and palate, defect, digital altered cast, digital dentistry, intraoral scanner, maxillofacial prosthetics, removable prosthesis, socket splicing method

## Abstract

In the prosthetic treatment of cleft lip and palate, there is a risk of material impaction, aspiration, or mis-swallowing due to the complex maxillary morphology and compromised pharyngeal function. Moreover, expertise is required to ensure the prosthesis harmonizes with the patient’s facial esthetics. Recent advancements in digital dentistry offer an effective and safer alternative to conventional methods. In this report, we describe a technique for fabricating a removable partial denture using a combination of digital devices, including an intraoral scanner, model scanner, 3D printer, and milling machine. A 59-year-old woman with a history of left-maxillary cleft lip and palate was treated with a digitally designed and fabricated prosthesis. The technique incorporated novel steps such as a digital altered cast technique and socket splicing method, allowing for improved functional adaptation and aesthetic integration by referencing 3D facial scans. This digital approach eliminated the need for traditional impression materials, reduced the number of clinical visits and adjustment sessions, and contributed to enhanced patient safety and satisfaction.

The aim of this report is to demonstrate the clinical application and advantages of digital technology in the fabrication of a removable prosthesis for a patient with cleft lip and palate.

## Introduction

Patients with cleft lip and palate often require prosthetic dental treatment due to congenital maxillary defects, including missing teeth and maxillary hypoplasia [1]. Although replacement of individual teeth using a dental implant prosthesis or fixed prosthesis is often done in cases with minor defects [2], the use of a removable prosthesis is still necessary in some cases, especially those with a larger defect and discrepancy [3-5]. Conventional methods for fabricating a prosthetic appliance involve the use of impression materials to obtain dental impressions. However, in patients with cleft lip and palate, there is an increased risk of material impaction, particularly due to the presence of fistulas and the complexities of the maxillary morphology. There is also a risk that compromised pharyngeal function could lead to mis-swallowing or aspiration of dental materials.

Since Patzelt et al. introduced the use of intraoral scanners for digitizing edentulous jaws, significant advancements have been made in the application of digital technology in maxillofacial prosthetics [6]. Elbashti et al. demonstrated the accuracy of digitizing jaw models in maxillectomy cases [7,8]. As Zhang et al. noted the challenges of capturing maxillary defects using intraoral scanners, alternative approaches have been explored [9]. One such method, which utilizes a pre-existing prosthesis as a reference for digital impression, was introduced by Towithelertkul et al. [10].

In the context of cleft lip and palate patients, Zhang et al. employed digital technology to evaluate long-term changes in dentition during prosthodontic rehabilitation [11]. Okazaki et al. reported the application of digital impression techniques in these patients, comparing their efficacy to that of conventional methods [12].

Digital technology is not limited to capturing intraoral structures; recording facial morphology also plays a critical role in prosthetic treatment. Several studies have reported on the use of digital technology for fabricating facial prostheses [13,14], while others have examined the impact of intraoral prostheses on facial surface contours [15,16]. Recently, 3D analysis has been effectively applied to evaluate the facial morphology of cleft lip and palate patients [17]. In such cases, the aesthetic appearance of the face in relation to the prosthesis must also be taken into consideration.

In the present case, we demonstrate a method for denture fabrication using digital devices including an intraoral scanner, model scanner, 3D printer, and milling machine.

## Technical report

**Figure 1:**
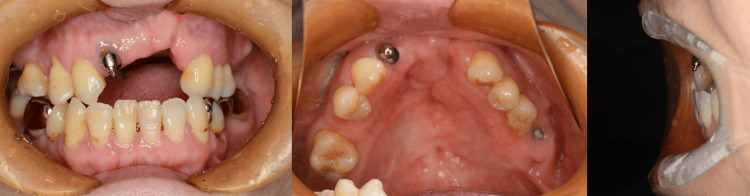
Intraoral views at the first consultation to our clinic.

The patient was a 59-year-old woman who visited our maxillofacial prosthetics clinic for continuous dental treatment (Figure 1). She had dental caries in the remaining dentition, specifically in the mandibular left first canine (tooth #33) and first premolar (tooth #34), and was experiencing complications from an ill-fitting denture. She was born with a left-maxillary cleft lip and palate and underwent lip repair surgery, palatoplasty, and subsequent orthodontic treatment. She also received a speech aid prosthesis and speech therapy.

She had been using an acrylic-resin-based partial denture equipped with O-ring attachments that was fabricated after she underwent pharyngeal flap surgery 20 years earlier. A treatment plan including re-fabrication of the prosthesis was developed. The prosthesis was designed with a metal framework, an acrylic resin base, and artificial teeth (#15, #12, #11, #21, #22, and #26). The metal framework included Akers clasps on teeth #16, #14, #23, #25, and a reinforcement line. The plan was explained to the patient and her consent was obtained. Comprehensive treatment was provided for both caries and periodontal disease in collaboration with a local practitioner of general dentistry.

**Figure 2:**
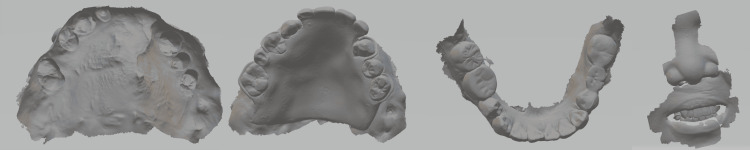
Intraoral and extraoral scan obtained using an intraoral scanner.

**Figure 3:**
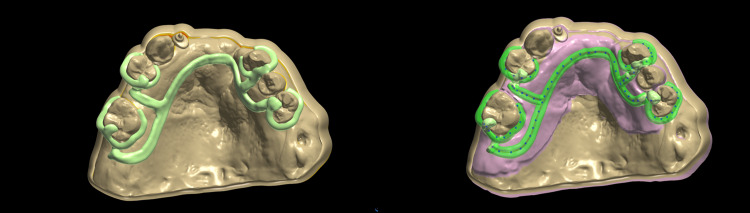
Design of the metal frame and denture base.

**Figure 4:**
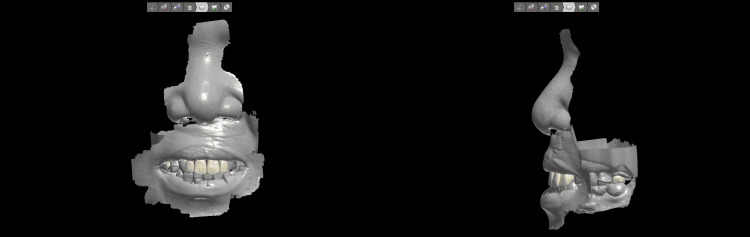
Design of the alignment of the front teeth with reference to the perioral scan.

**Figure 5:**
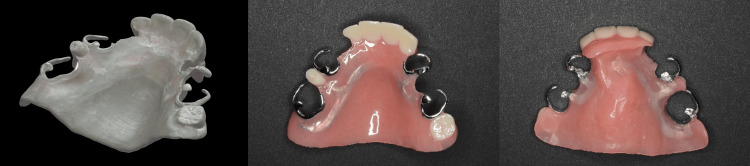
Scan of the wax try-in denture adjusted by palatogram and the completed definitive prosthesis.

**Figure 6:**
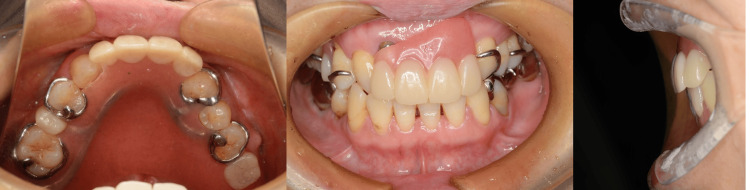
Intraoral views with the new prosthesis in place.

The new prosthesis was fabricated by incorporating digital technologies as described in the following technique:

1. Adjust the pre-existing denture using soft relining material (Coe-soft, GC Corporation, Tokyo, Japan).

2. Scan the intaglio and polished surface of the adjusted pre-existing denture using a model scanner (D2000 Lab Scanner, 3Shape, Copenhagen, Denmark).

3. Scan the oral mucosal surfaces and the remaining dentition with and without wearing the pre-existing denture using an intraoral scanner (TRIOS 3 Intraoral Scanner, 3Shape, Copenhagen, Denmark) (Figure 2).

4. Scan the perioral facial regions with the intraoral scanner (Figure 2).

5. Superimpose all the 3D data using dental computer-aided design software (3Shape Dental System, 3Shape, Copenhagen, Denmark) and merge the data using general-purpose 3D modeling software (Meshmixer, Autodesk, San Francisco, CA).

6. Design the prosthesis including the metal frame design using the superimposed digital data in dental computer-aided design software (Figure 3), with the arrangement of anterior artificial teeth determined in reference to the perioral facial region data (Figure 4).

7. Print out the metal frame design in castable material (Castable Wax Resin, Formlabs, Somerville, MA) with a dental 3D printer (Form 3B+, Formlabs, Somerville, MA), and cast a cobalt-chromium frame.

8. Fabricate the denture base with acrylic resin (Surgical Guide Resin, Formlabs, Somerville, MA) via 3D printing, with the artificial teeth section produced by a milling process (DWX-52DC, DGSHAPE, Hamamatsu, Japan) using wax (Wax Disk, Yamahachi Dental, Gamagori, Japan).

9. Assemble these components, and then try and fit them in the patient. Adjust the trial denture by adding soft wax (Soft Plate Wax, GC Corporation, Tokyo, Japan) according to the result of a palatogram using powder of irreversible hydrocolloid impression material (Hi-Technicol, GC Corporation, Tokyo, Japan).

10. Scan the adjusted trial denture (Figure 5), adjust the final design, and splice it into parts using the general-purpose 3D modeling software. With the data, mill the base using auto-polymerizing denture base acrylic resin (Palapress vario, Kulzer, Hanau, Germany) and mill the teeth using high-grade hybrid resin (Shofu Disc HC, Shofu, Kyoto, Japan).

11. Assemble all the parts using the denture base acrylic resin (Figure 5) and deliver the denture to the patient (Figure 6), confirm its fit and function, and adjust it accordingly.

The schedule and number of visits needed for the fabricated prosthesis in the method presented in this study and in conventional fabrication are described in Table 1. The prosthesis was fabricated with fewer visits compared with the conventional method. It functioned well after fewer adjustments. The patient was satisfied with the denture and transitioned relatively quickly into the maintenance phase.

**Table 1:** Details of treatment and number of visits for adjustments needed.

	Conventional prosthesis	Prosthesis fabricated in digital technique
Fabrication steps	1. Preliminary impression	1. Intraoral and denture scanning
	2. Definitive impression	2. Pre-treatment, scan refinement
	3. Bite registration	3. Trial fit and adjustments
	4. Trial fit and adjustments	4. Denture delivery and post-delivery instructions
	5. Denture delivery and post-delivery instructions	-
Adjustment count	6	1

## Discussion

In this case, acrylic resin was selected as the base material. A metal base such as cobalt chromium base would be another option. On the maxilla, a metal base would have the benefit of making the base thin. However, in the present case, a maxillary defect was present due to a congenital deformity and the thickness of the denture was important to compensate for the defect [1]. In this situation, making a thin prosthesis using a metal base would not be beneficial. In addition, the defective part of the denture sometimes requires more adjustment compared with other parts. Acrylic resin is compatible with such adjustment, leading to the selection of the acrylic resin base in this case.

Technique #5 is named the “digital altered cast technique.” It involves digitally stitching intraoral scan data of the teeth with the intaglio surface of a well-fitting denture. This approach is conceptually similar to the altered cast technique used in conventional workflows. Technique #10 is named the “socket splicing method.” It involves digitally splitting the prosthesis into separate components: the denture base, divided into several parts, the artificial teeth, and the metal framework. This segmentation allows the base to be milled without undercuts, which are then precisely assembled with the artificial teeth and the metal framework. Using these two newly named techniques, the prosthesis was successfully designed and fabricated based on digital 3D data. These techniques allowed for significant improvements in both the accuracy and functional reproduction of the prosthesis compared to conventional digital workflows, such as direct mucosal surface scanning. Specifically, technique #5 facilitated the integration of functional impression data, resulting in a prosthesis that more closely replicated the patient's dynamic oral conditions, while technique #10 enabled the precise assembly of digitally milled components. Fabrication in this way circumvented the use of conventional impression materials and reduced the number of patient visits (Table 1). Only one visit was needed for the adjustment because of the sufficient fit of the prosthesis. With the new techniques, the patient needed one fewer visit for fabrication and five fewer visits for adjustment compared to the conventional method. In conventional jaw relation recording, reference lines such as the midline and lip line are marked on the occlusion rim to guide tooth placement and esthetics. In the new technique, referencing data from perioral facial regions facilitated a comprehensive three-dimensional evaluation, which was crucial in achieving optimal aesthetic rehabilitation. Detailed attention to facial contours and structures in the final prosthetic design not only ensured functional rehabilitation but also achieved harmonious alignment with the patient’s facial aesthetics.

The application of digital technology in the prosthetic dental treatment of patients with cleft lip and palate offers distinct advantages. It not only eliminates risks associated with impaction of impression materials, mis-swallowing, and aspiration but also significantly benefits aesthetic rehabilitation [12,17]. Additionally, this technique may enable dental practitioners who are less experienced in maxillofacial prosthetics to attempt the fabrication of prostheses for patients with maxillofacial defects. In this case, the amount of undercut was precisely calculated, and based on this, the clasp was automatically designed. However, the clasp extended into the aesthetic zone, making it an unfavorable design from an esthetic perspective. Due to the limited vestibular depth caused by cleft lip and palate surgery, the maxillary anterior teeth were not prominently visible, so the esthetic issue was minimal in this case. Nevertheless, this is an important point to consider when using automated design in the future.

Despite the advantages demonstrated in this case, there are several limitations to the presented technique. First, this digital workflow relies on the presence of a pre-existing denture, which serves as a reference for capturing functional morphology and occlusion. In cases where no previous prosthesis is available, alternative methods or conventional techniques may still be required. Second, the implementation of this technique requires access to multiple digital dental devices. The high cost of these devices may limit their availability and widespread adoption in general clinical settings. Third, since digital dental devices are primarily designed for crown-bridge prostheses and implant prosthetics, their effective use in removable prosthesis fabrication requires innovation. The successful fabrication of such a prosthesis depends on the skills of an experienced dental technician. Continued exploration and refinement of these methods remain imperative in the future.

## Conclusions

The use of digital techniques to fabricate a removable prosthesis for a patient with cleft lip and palate was described in this report. The prosthesis was successfully and safely fabricated, avoiding the risk of impaction, mis-swallowing, and aspiration of impression material. The required number of patient visits was also reduced. By scanning the perioral facial region, aesthetic rehabilitation was also achieved. The application of digital technology in prosthetic dental treatment for patients with cleft lip and palate offers distinct advantages.
